# Neurobehavioral recovery in patients who emerged from prolonged disorder of consciousness: a retrospective study

**DOI:** 10.1186/s12883-020-01758-5

**Published:** 2020-05-20

**Authors:** Hoo Young Lee, Jung Hyun Park, Ae Ryoung Kim, Misun Park, Tae-Woo Kim

**Affiliations:** 1 TBI rehabilitation center, National Traffic Injury Rehabilitation Hospital, 260, Jungang-ro, Dogok-ri, Yangpyeong-eup, Yangpyeong-gun, Gyeonggi-do 12564 South Korea; 2 Department of Rehabilitation Medicine, Seoul National University Hospital, Seoul University College of Medicine, Seoul, South Korea; 3 Department of Medicine, the Graduate School of Yonsei University, Seoul, South Korea; 4 Department of Rehabilitation Medicine, Gangnam Severance Hospital, Rehabilitation Institute of Neuromuscular Disease, Yonsei University College of Medicine, Seoul, South Korea; 5 Department of Rehabilitation Medicine, School of Medicine, Kyungpook National University, Kyungpook National University Hospital, Daegu, South Korea; 6 Department of Biostatistics, Clinical Research Coordinating Center, Catholic Medical Center, The Catholic University of Korea, Seoul, Republic of Korea

**Keywords:** Brain injuries, Consciousness disorders, Rehabilitation, Treatment outcome

## Abstract

**Background:**

We investigated the clinical course of patients with prolonged disorders of consciousness (PDoC), predictors of emergence from PDoC (EDoC), and the temporal dynamics of six neurobehavior domains based on the JFK Coma Recovery Scale-Revised (CRS-R) during the recovery.

**Methods:**

A total of 50 traumatic and non-traumatic patients with PDoC were enrolled between October 2014 and February 2017. A retrospective analysis of the clinical findings and neurobehavioral signs was conducted using standardized methodology such as CRS-R. The findings were used to investigate the incidence and predictors of EDoC and determine the cumulative pattern of neurobehavioral recovery at 6 months, 1 year, and 2 years post-injury.

**Results:**

The results showed that 46% of the subjects emerged from PDoC after 200 median days (64–1197 days) of injury onset. The significant predictors of EDoC included minimally conscious state (MCS) (vs. vegetative state), higher auditory, communication, arousal, total CRS-R scores, shorter lag time post-injury, and the absence of intra-axial lesions. In terms of cumulative recovery of motor and communication signs in patients who emerged from PDoC, 39 and 32% showed EDoC at 6 months post-injury, and 88 and 93% exhibited EDoC at 2 years post-injury, respectively.

**Conclusions:**

Nearly half of the patients with PDoC recovered consciousness during inpatient rehabilitation. MCS, shorter lag time, the absence of intra-axial lesions, higher auditory, communication, arousal, and total CRS-R scores were important predictors for EDoC. Motor scores in the early stage of recovery and communication scores after prolonged intervals contributed to the higher levels of cumulative EDoC.

## Background

Disorders of consciousness (DoC), including vegetative state/unresponsive wakefulness syndrome (VS/UWS) and minimally conscious state (MCS), indicate a continuum of disruption in the arousal and awareness systems of the brain caused by severe acquired brain injury (ABI) [1–4]. VS/UWS is characterized by a lack of response to the environment, but spontaneous eye-opening along with evidence of sleep-wake cycles. In contrast, patients in MCS may demonstrate inconsistent but reproducible signs of awareness. Patients with prolonged DoC (PDoC) remain in VS/UWS or MCS for more than 4 weeks [5]. The US Aspen Workgroup proposed that emergence from DoC is characterized by reliable and consistent displays of functional communication with or without the functional use of objects [1–4].

The number of studies regarding the natural course of DoC after an ABI has grown over more than the last decade. Specifically, those with traumatic etiologies and diagnosis of MCS (as opposed to VS/UWS) at the time of rehabilitation admission have shown better prognoses, with regard to both the recovery of consciousness and the recovery of functional independence [6–9].

Even though studies have begun to demonstrate the recovery potential in certain subsets of patients with DoC, the outcomes and conclusions are comprehensibly heterogeneous across studies with rates of recovery of consciousness that range from 14 to 95% [7]. Moreover, the nature, features, and prediction of the recovery process have not been fully elucidated.

These factors emphasize the need to investigate the clinical course and neurobehavioral recovery in patients who have emerged from DoC. Enhanced knowledge regarding the long-term outcome of individuals with PDoC may help clarify the range of outcomes expected after severe ABI and guide treatment decisions that reflect a more accurate assessment of patient prognosis.

It is very important to recognize changes and predict recovery from VS/UWS and MCS to emergence from PDoC in severely brain-injured patients who may be expected to survive their initial brain insults and transition through various states of impaired consciousness [10, 11]. It is especially important to understand the nature and course of neurobehavioral recovery based on the overall and hierarchical perspectives.

The aim of this study was to investigate the course and clinical characteristics of patients emerging from PDoC during neurorehabilitation and present a predictive model for the recovery of consciousness. In terms of tracking serial changes in the JFK Coma Recovery Scale-Revised (CRS-R), this study was the first of its kind to investigate the temporal dynamics of each neurobehavioral sign in CRS-R and their effects on the emergence from PDoC.

## Methods

The study was a retrospective, observational study of patients with PDoC who were admitted to a comprehensive neurorehabilitation hospital in the Republic of Korea over a 3-year period from October 1, 2014, to February 28, 2017. The inpatient rehabilitation of patients with PDoC in Korea entails intense rehabilitative treatment for at least 3 hours each day during the first 2 years after onset, and about an hour and a half thereafter.

We retrospectively collected data from the Clinical Data Warehouse (CDW) in the hospital, including a database of electronic medical records obtained from both inpatients and outpatients for real-time clinical analysis of the raw data with the approval of the Institutional Review Board of the National Traffic Injury Rehabilitation Hospital (No. NTRH-18005). The CDW contains almost all the medical records, including every field note of the medical staff (admission and discharge notes, progress reports, and nursing data), patient information data, and records (insurance, diagnostic codes, age, gender, and vital signs), test results (laboratory tests, functional assessments, and imaging studies) and treatment modalities (medications, therapies, and medical procedures). The IRB, in accordance with the Declaration of Helsinki, approved this study and granted waiver of consent because the data had been de-identified before they were used for the analysis of this study.

**Fig. 1:**
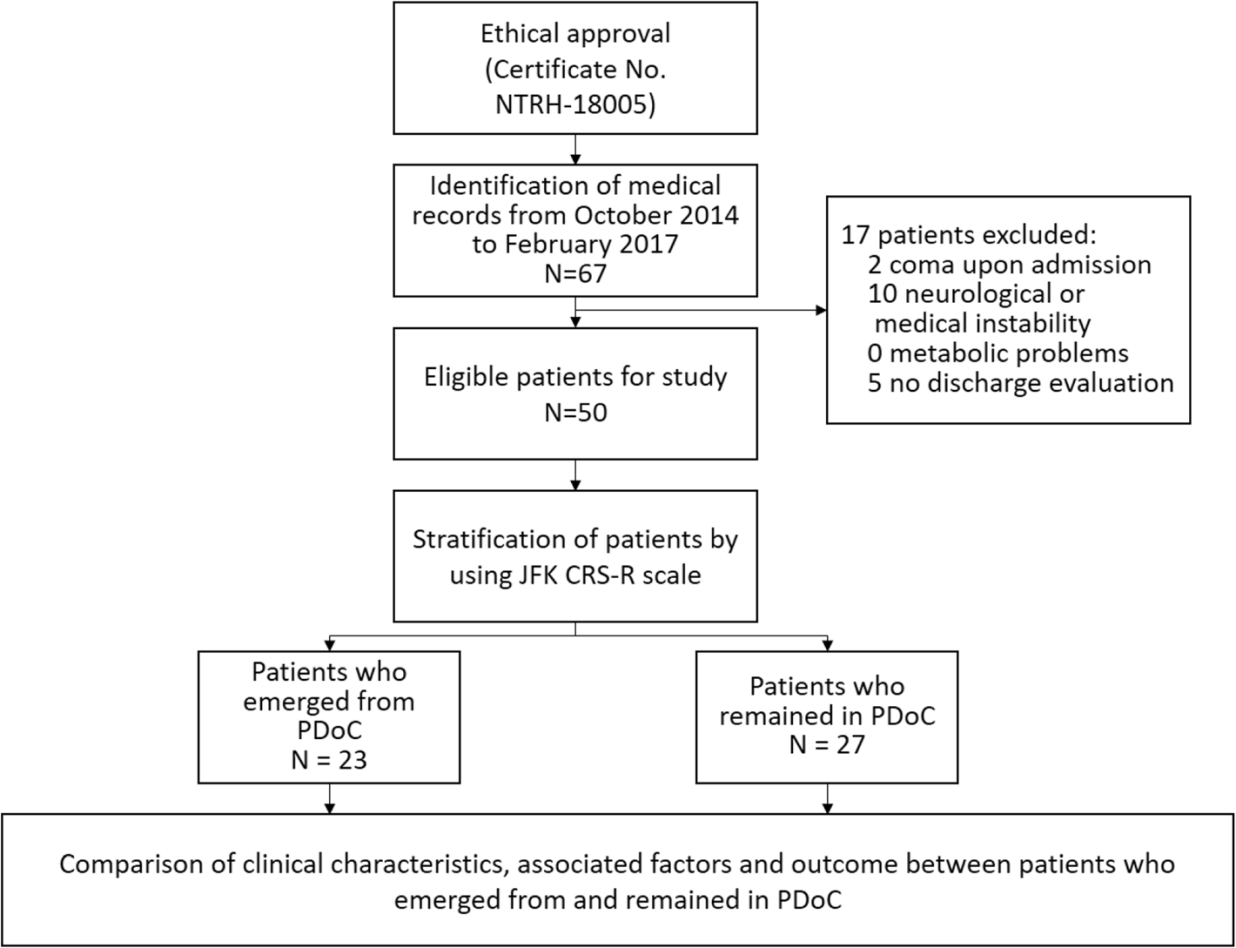
Patient recruitment and retrospective protocol methodology. CRS-R, JFK Coma Recovery Scale-Revised; PDoC, prolonged disorder of consciousness

The inclusion criteria were patients of all ages with acquired traumatic or non-traumatic brain injury who were diagnosed with VS/UWS or MCS upon admission and based on serial evaluation data acquired during hospitalization. We confirmed the clinical diagnosis of VS/UWS, MCS, and EDoC based on CRS-R scores. Patients diagnosed with coma upon admission, exhibiting neurological or medical instability, and those without discharge evaluation were excluded. Patients with metabolic problems, which may provoke decreases in the level of consciousness, were also excluded (Fig. 1).

Even with various milestones of impaired consciousness, the patients were dichotomized into two groups depending on the emergence from PDoC during rehabilitation or persistent VS/UWS or MCS upon discharge.

During hospitalization, all patients underwent standardized and serial clinical evaluations for behavioral responsiveness. All patients were assessed with CRS-R upon admission and at a predetermined time, by a well-trained expert team composed of rehabilitation physicians and physical and occupational therapists who had more than 1 year of experience in evaluations. The evaluation of CRS-R is based upon specific behavioral responses to sensory stimuli on 30 hierarchically arranged items administered in a standardized format. The lowest item on each subscale represents reflexive activity, whereas the highest items represent cognitive behaviors [12].

To determine the most consistent states of consciousness and rehabilitation outcomes, none of the centrally acting pharmacologic agents administered daily, such as antispasmodics, anticonvulsants, and neurostimulatory agents were withdrawn.

Along with pharmacological interventions, all patients received physical therapy and occupational therapy in a neurorehabilitation program for 3 h a day, 5 days a week. Whole-body vibration, neuromuscular electric stimulation, Bobath, kinesthetic stimulation, joint movement and range of motion exercise, mobility management, and tilt-table standing were provided by the physical therapists. Multisensory stimulation, sensory regulation or basal stimulation, familiar auditory sensory training and facio-oral stimulation techniques were provided by the occupational therapists. In all cases, the data were entered prospectively into the CDW because they were standardized test results. All the evaluators were blinded to the data used for advanced retrospective analysis.

After dividing the patients into two groups, the baseline characteristics, admission CRS-R scores, and 12 predictor variables associated with the incidence of consciousness recovery were investigated. The independent variables were as follows: 1) sex, 2) age at injury onset, 3) level of consciousness at admission (VS/UWS or MCS), 4) cause of the ABI (traumatic brain injury (TBI) or non-TBI), 5) the injury type (extra-axial or intra-axial lesion), 6) the lag time from the injury, 6) the CRS-R score at admission, 7) hydrocephalus, 8) ventriculoperitoneal shunt, 9) cranioplasty, 10) treatment with anticonvulsants (continued or discontinued), 11) seizure events, and 12) the level of education (< 12 years or ≥ 12 years). Further, we compared the degree of advancement in each sign of CRS-R during inpatient rehabilitation. Finally, we analyzed the temporal dynamics of auditory, visual, motor, oromotor, communication, and arousal scores and compared their effects on neurobehavioral recovery.

The baseline differences between the two subgroups were analyzed by the Wilcoxon rank-sum test for continuous and ordinal variables, and the chi-squared test or Fisher’s exact test for categorical variables. The predictors of EDoC were analyzed by the univariate Cox proportional hazards model. The adjusted multivariate Cox proportional hazards model was used to investigate the optimal prediction parameters for EDoC. Maximally selected rank statistics were used to estimate the optimal cutoff value.

Kaplan–Meier plots were used to identify the median days at which each subscale of the CRS-R showed improvement of 1 point or more, and motor and communication scores reflecting EDoC. The plots were then converted to cumulative probabilities of attaining at least 10% progress in each sign and emergence in the motor and communication subscale at 6 months, 1 year, and 2 years post-injury.

Prognostic correlation between the CRS-R subscale scores and EDoC was analyzed using the marginal structural Cox model, after adjustment for time-varying confounders, such as clustered data on CRS-R at various time points from injury, during the longitudinal observation period.

Statistical analysis was performed using R software (version R.3.3.2; the R Foundation) and SAS version 9.4.

## Results

### Patient clinical demographics

Among the total of 1236 inpatients monitored during the 3-year observation period, 40.9% (*n* = 506) had acquired brain injuries as their main diagnosis. Of those patients, 13.2% (*n* = 67) were diagnosed with PDoC and 9.9% (*n* = 50) referred for further analysis (Fig. 1) (Supplementary file 1).

The patients progressed through the stages of recovery at varying rates. Of the 50 patients, 25 were admitted with VS/UWS. Among the 12 VS/UWS patients who showed improvement in the level of consciousness, eight recovered to the MCS level and four demonstrated EDoC. Of the 25 patients who were admitted in MCSs, 19 emerged from an MCS. Overall, 46% emerged from PDoC during inpatient rehabilitation. During the observational period, no patients died and no patient was lost to follow-up. The stimulant medications prescribed to the patients are summarized in Table 1.

**Table 1:** Summary of neuroplasticity stimulant drugs given to the patients with PDoC

Prescribed Drugs	EDoC (*n* = 23)	PDoC (*n* = 27)
Noradrenergic	9	13
atomoxetine		
Dopaminergic	18	14
levodopa/carbidopa		
methylphenidate		
Cholinergic	26	17
choline alfoscerate		
donepezil		
rivastigmine		
Serotonergic	9	3
escitalopram		
paroxetine		
sertraline		
Glutamatergic	3	2
memantine		
Others	9	6
nicergoline		
oxiracetam		
zolpidem		

*EDoC* emergence from disorder of consciousness, *PDoC* prolonged disorder of consciousness

The median (IQR) lag time from injury was 204.5 (97.25, 374.5) and the duration of inpatient rehabilitation was 92 (62.5, 121) days. In the recovery group, the emergence from PDoC occurred over a median period of 200 (129.5, 329, range 64–1197) days. Stratification of the recovery group based on diagnostic subtype (VS/UWS vs. MCS) indicated that patients with VS/UWS and MCS manifested EDoC in 164 (124, 236.25, range 112–345) and 209 (131.5, 346, range 64–1143) median days, respectively. Sub-group analysis of the participants by etiology (traumatic vs. non-traumatic) revealed that the patients with traumatic injury regained consciousness over a period of 158 (124.25, 292.5, range 85–575) median days, and a median of 217 (154, 345, range 64–1143) days for non-traumatic injuries.

To investigate the prognostic outcomes in PDoC, the patients were retrospectively dichotomized into patients who emerged from PDoC and those who remained in PDoC states. Based on descriptive analysis, MCS (76% vs. 24%, *p* <  0.001), greater total CRS-R scores (12.6 ± 3.8 vs.6.1 ± 3.8, p <  0.001), extra-axial hemorrhage compared with intra-axial lesions (87.5% vs. 12.5%, *p* = 0.014), and shorter lag time from injury (219.1 ± 232.3 days vs. 321.5 ± 266.2 days, *p* = 0.048) were associated with emergence from PDoC (Table 2).

**Table 2:** Predictors of emergence from disorder of consciousness in univariate analysis

Variable	EDoC (*n* = 23)	PDoC (*n* = 27)	*p-*value^†^	HR (95% CI)^‡^	*p-* value^‡^
Sex					
Male	16 (50)	16 (50)	0.645	reference	
Female	7 (38.9)	11 (61.1)		0.44 (0.17, 1.15)	0.093
Age					
Median (IQR)	46 (34, 62.5)	46 (20, 60)	0.326	1.02 (0.99, 1.04)	0.115
Level of consciousness					
VS/UWS	4 (16)	21 (84)	< 0.001	reference	
MCS	19 (76)	6 (24)		4.49 (1.52, 13.27)	0.007
Total CRS-R score					
Median (IQR)	13 (10,16)	5 (4, 7.5)	< 0.001	1.16 (1.06, 1.28)	0.002
≤ 6*	1 (5.6)	17 (94.4)		reference	
> 6	22 (68.8)	10 (31.2)		10.02 (1.34, 74.61)	0.028
Etiology					
TBI	14 (48.3)	15 (51.7)	0.927	reference	
Non-TBI	9 (42.9)	12 (57.1)		0.8 (0.3, 1.9)	0.61
Injury Type (*n* = 46)					
Extra-axial hemorrhage	7 (87.5)	1 (12.5)	0.014	reference	
Intra-axial lesion	13 (34.2)	25 (65.8)		0.09 (0.03, 0.24)	< 0.001
Lag Time (days)					
Median (IQR)	133 (86, 212.5)	222 (126, 443.5)	0.048	0.99 (0.98, 0.99)	< 0.001
≤ 528*	21 (48.8)	22 (51.2)		reference	
> 528	2 (28.6)	5 (71.4)		0.10 (0.01, 0.78)	0.028
Hydrocephalus					
Present	11 (40.7)	16 (59.3)	0.6	reference	
Absent	12 (52.2)	11 (47.8)		1.77 (0.78, 4.06)	0.174
VP shunt					
Present	8 (50)	8 (50)	0.932	reference	
Absent	15 (44.1)	19 (55.9)		1.28 (0.54, 3.04)	0.573
Cranioplasty					
Present	10 (43.5)	13 (56.5)	0.964	reference	
Absent	13 (48.1)	14 (51.9)		1.69 (0.73, 3.91)	0.218
Anticonvulsants					
Continued	15 (45.5)	18 (54.5)	> 0.999	reference	
Discontinued/not taking	8 (47.1)	9 (52.9)		1.19 (0.5, 2.86)	0.694
Education (*n* = 19)					
< 12 yrs	3 (25)	9 (75)	0.156	reference	
≥ 12 yrs	20 (54.1)	17 (45.9)		1.95 (0.58, 6.6)	0.282

Values are presented as median (IQR) or number (%)

*CRS-R* JFK Coma Recovery Scale-Revised, *VS/UWS* vegetative state/unresponsive wakefulness syndrome, *MCS* minimally conscious state, *EDoC* emergence from disorder of consciousness, *PDoC* prolonged disorder of consciousness, *TBI* traumatic brain injury, *HR* hazard ratio

^*^The optimal cutoff values of each variable were determined by maximally selected log-rank statistics

^†^*P*-value for the difference was determined by chi-squared, Fisher’s exact, the Wilcoxon rank-sum tests

^‡^Hazard ratio and *p*-value were calculated by univariate Cox proportional hazards regression

In terms of CRS-R scores, the admission scores on the auditory (2.3 ± 0.9 vs. 1.1 ± 1.0, p <  0.001), visual (2.7 ± 1.3 vs. 1.0 ± 1.3, p <  0.001), motor (3.4 ± 1.4 vs. 1.5 ± 1.1, *p* < 0.001), oromotor (1.3 ± 0.7 vs. 0.7 ± 0.7, *p* = 0.002), communication (0.7 ± 0.5 vs. 0.1 ± 0.3, p < 0.001), and arousal (2.1 ± 0.7 vs. 1.7 ± 0.7, *p* = 0.046) subscales were significantly higher in patients who emerged from PDoC compared with those who remained in PDoC states. Further, the degree of advancement in each CRS-R subscale during neurorehabilitation was significantly greater in the patients who emerged from PDoC compared with those who remained in PDoC states (Table 3) (Supplementary file 2).

**Table 3:** Descriptive data for progress in CRS-R scores during neurorehabilitation

Outcome Measures	Emergence from PDoC		*p*-value^*^	Remain as PDoC		*p*-value^*^	*p*-value^†^	*p*-value^‡^
	Admission	Discharge		Admission	Discharge			
Auditory	2 (2, 3)	4 (3.5, 4)	< 0.001	1 (1, 1)	1 (1, 2)	0.042	< 0.001	< 0.001
Visual	3 (2, 4)	4 (4, 5)	< 0.001	1 (0, 1)	1 (1, 3)	0.011	< 0.001	0.004
Motor	4 (2, 5)	6 (5, 6)	< 0.001	2 (1, 2)	2 (1, 3)	0.005	< 0.001	0.001
Oromotor	1 (1, 2)	2 (2, 3)	0.001	1 (0, 1)	1 (1, 1)	0.001	0.002	0.041
Communication	1 (0, 1)	2 (2, 2)	< 0.001	0 (0, 0)	0 (0, 0)	0.149	< 0.001	< 0.001
Arousal	2 (2, 2.5)	3 (3, 3)	< 0.001	2 (1, 2)	2 (2, 2)	0.105	0.046	0.001

Values are presented as median (IQR)

*CRS-R* JFK Coma Recovery Scale-Revised, *PDoC* prolonged disorder of consciousness

^*^ Comparison between CRS-R scores at admission and discharge in each group

^†^ Comparison between admission CRS-R scores in the dichotomized groups

^‡^Comparison between the degrees of advancement in the CRS-R scores in the dichotomized groups

### Optimal outcome prediction: variables and models

Cox regression analysis was performed to investigate the significant predictors of emergence from PDoC. MCS and higher CRS-R scores at admission were significantly correlated with positive outcomes, whereas intra-axial brain lesions and prolonged lag time were significant predictors of negative outcomes (Table 1).

According to the multivariate Cox regression analysis and Akaike information criterion-based optimization, lag time and intra-axial lesions were significantly negatively correlated with emergence from PDoC.

In terms of the optimal cutoff value, lag days from the injury onset to neurorehabilitation within 528 days and total CRS-R scores greater than 6 were significantly associated with emergence from PDoC.

Based on the neurobehavioral level upon admission, all the subscale scores in CRS-R significantly affected the emergence from PDoC. The arousal, communication, and auditory subscales were strongly correlated with emergence from MCS, followed by oromotor, visual, and motor subscales. The total CRS-R scores had the least impact (Table 4).

**Table 4:** CRS-R variables as predictors of emergence from disorder of consciousness

Variable	EDoC (*n* = 23)	PDoC (*n* = 27)	HR (95% CI)	*p*-value
Auditory				
Median (IQR)	2 (2, 3)	1 (1, 1)	9.5 (5.59, 16.14)	< 0.001
Visual				
Median (IQR)	3 (2, 4)	1 (0, 1)	3.78 (2.92, 4.90)	< 0.001
Motor				
Median (IQR)	4 (2, 5)	2 (1, 2)	3.29 (2.23, 4.85)	< 0.001
Oromotor				
Median (IQR)	1 (1, 2)	1 (0, 1)	4 (2.35, 6.81)	< 0.001
Communication				
Median (IQR)	1 (0, 1)	0 (0, 0)	11.01 (6.35, 9.09)	< 0.001
Arousal				
Median (IQR)	2 (2, 2.5)	2 (1, 2)	22.4 (6.34, 79.12)	< 0.001
Total score				
Median (IQR)	13 (10, 16)	5 (4, 8)	1.59 (1.41, 1.8)	< 0.001

Values are presented as median (SD) or number (%)

*CRS-R* JFK Coma Recovery Scale-Revised, *EDoC* emergence from prolonged disorder of consciousness, *PDoC* prolonged disorder of consciousness, *HR* hazard ratio

### Temporal dynamics of neurobehavioral signs during emergence from PDoC

The median number of days required to advance at least one point in each CRS-R subscale was determined from Kaplan-Meier curves for the groups that emerged from PDoC. Among various recovery patterns, motor signs showed the most rapid recovery (191 median days), followed by auditory, arousal, communication, and visual scores in order. Oromotor scores showed the maximum improvement delay (284 median days) (Table 5).

**Table 5:** Median achievement time and cumulative probability to reach at least 1-point advancement

Outcome Measures	Emergence from PDoC (*n* = 23)					PDoC (*n* = 27)				
	Event	Median achievement time (95% CI, days)	Cumulative probability			Event	Median achievement time (95% CI, days)	Cumulative probability		
			180 days	365 days	730 days			180 days	365 days	730 days
Auditory	20	206 (127, 270)	0.37	0.76	0.95	7	NA (534, NA)	0.08	0.24	0.48
Visual	16	269 (127, 400)	0.42	0.61	0.92	10	614 (252, 614)	0.15	0.39	0.51
Motor	20	191 (123, 270)	0.43	0.76	0.93	12	534 (289, 614)	0.15	0.44	0.61
Oromotor	17	284 (154, 593)	0.36	0.61	0.76	11	614 (274, 1143)	0.08	0.36	0.56
Communication	14	239 (117, 406)	0.37	0.68	0.92	4	NA (534, NA)	0.04	0.14	0.26
Arousal	18	231 (127, 437)	0.38	0.65	0.90	5	NA (534, NA)	0.04	0.19	0.30

*PDoC* prolonged disorder of consciousness, *NA* not applicable

**Fig. 2:**
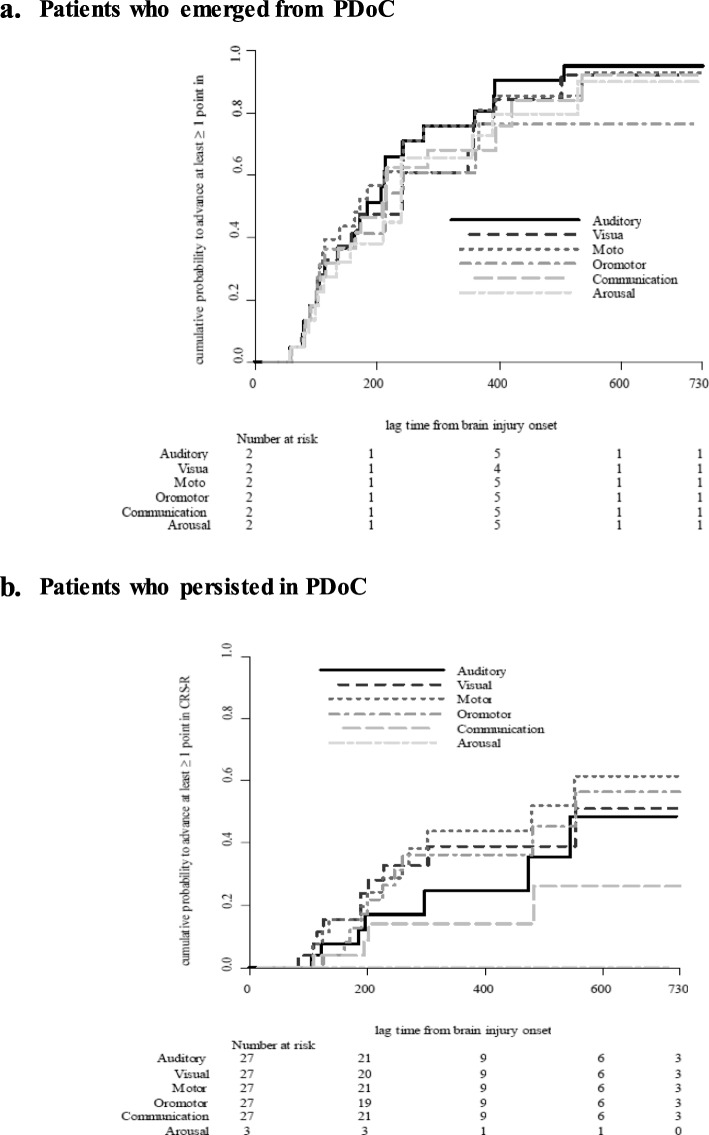
Cumulative probability to reach at least 1-point advancement in the CRS-R. **a** Patients who emerged from PDoC. **b** Patients who persisted in PDoC

Meanwhile, the cumulative probabilities of at least one point of progress across the full range of each subscale showed a diverse pattern depending on the stage of recovery. At 180 days post-injury, the greatest cumulative probability of advancing one or more points was observed in the motor (43%), visual (42%), arousal (38%), auditory and communication (37%, both), and oromotor (36%) scores. At 2 years post-injury, the auditory score showed the highest cumulative probability of 95%, followed by motor (93%), visual and communication (92% both), and arousal (90%) scores, with the least probability in oromotor scores (76%) (Table 5, Fig. 2).

**Fig. 3:**
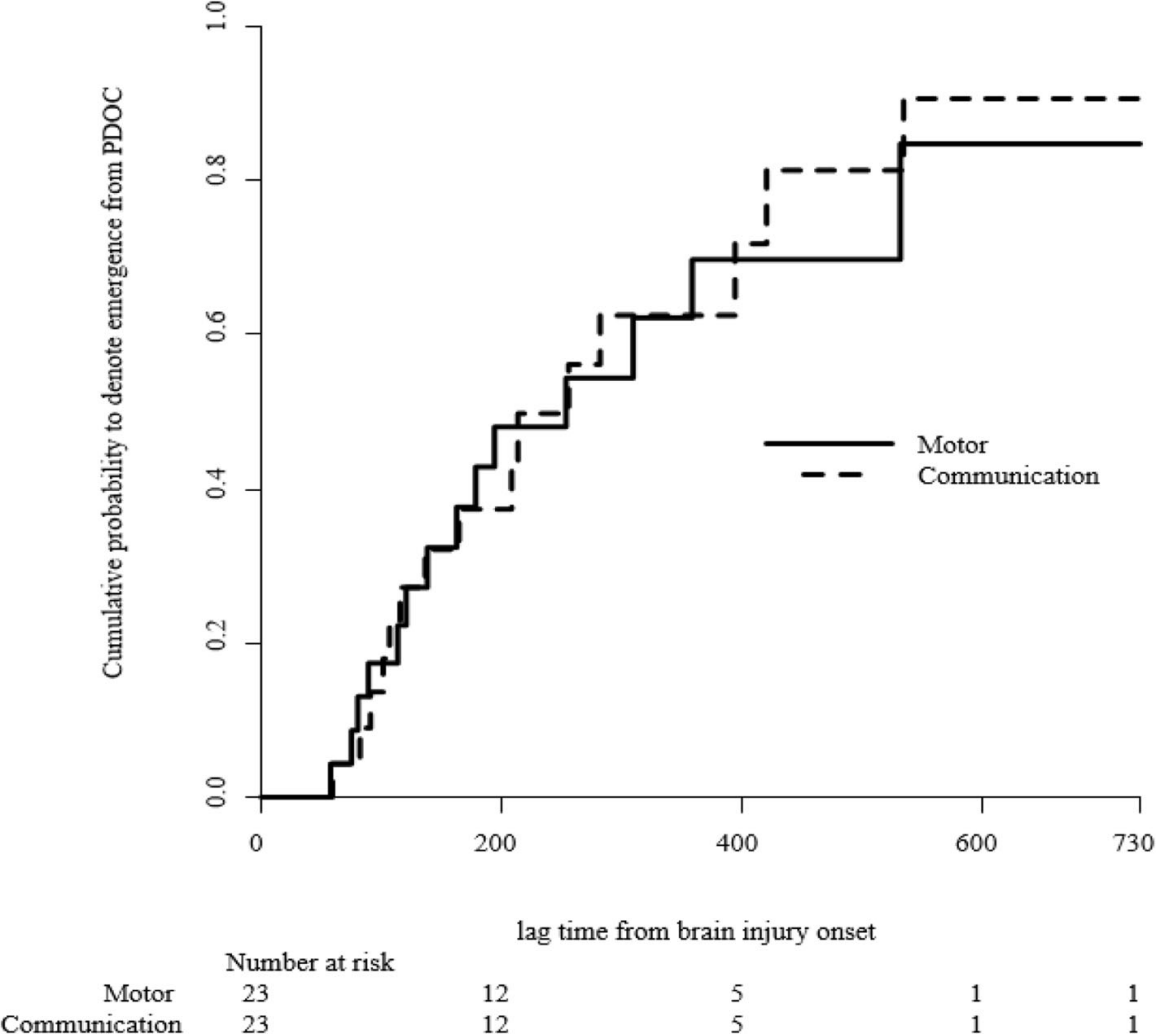
Cumulative probability of EDoC in the CRS-R subscales. During the 180-day post-injury period, 39 and 32% of the patients in the recovery group manifested EDoC in motor and communication skills, respectively. Over time, the cumulative EDoC in the communication subscale increased and exceeded the motor subscale at 284 days post-injury. At 2 years post-injury, 88 and 93% of patients in the recovery group manifested EDoC in motor and communication skills, respectively. CRS-R, JFK Coma Recovery Scale-Revised; EDoC, emergence from disorder of consciousness; PDoC, prolonged disorder of consciousness

We further investigated the temporal dynamics and cumulative probabilities of motor and communication scores associated with the following abilities: (1) functional use of objects, that is behavioral evidence of the ability to discriminate between at least two different objects and, (2) functional interactive communication, which may occur through verbalization, writing, ‘yes’ or ‘no’ signals, or the use of augmentative communication devices, which specifically correspond to EDoC (Fig. 3). Among 23 patients who manifested EDoC, 17 demonstrated EDoC via the functional use of objects at 209 (range 154–400) median days, whereas 18 showed EDoC via functional communication at 284 (range 150–390) median days. With regard to cumulative recovery, the functional use of objects was greater than the functional interaction at 180 days post-injury (32% vs. 39%). Eventually, the cumulative EDoC in the communication subscale increased and exceeded the motor subscale at 284 days post-injury. At 2 years post-injury, 93% of the recovery group showed functional interaction while 88% demonstrated the functional use of objects.

## Discussion

In this study, a retrospective observational analysis revealed a significant recovery of consciousness in patients with PDoC during inpatient rehabilitation, with 46% of the enrolled subjects emerging from PDoC. MCS, shorter lag time, the absence of intra-axial lesions, and higher auditory, communication, arousal, and total CRS-R scores were important predictors of EDoC. The model incorporating shorter lag time post-injury and the absence of intra-axial lesions best predicted the EDoC. The communication and auditory scores suggested a delayed but stronger correlation with EDoC compared with motor scores.

The strength of the study was that a wide range of clinical variables, including the whole subscales of CRS-R, were tracked longitudinally. In contrast to previous studies, we elucidated the course, predictive power, and effects of an extensive spectrum of neurobehavioral signs on the emergence from DOC, thus providing new insights into an optimal inpatient rehabilitation program that would best evaluate and maximize the potential for the recovery of consciousness. The merits of the methodology applied in our study were that the analysis of the full CRS-R performance profile, which includes all six subscale scores, enabled the accurate detection of conscious awareness [13]. Furthermore, our findings are supported by practice guidelines and updated recommendations for PDoC developed by the American Academy of Neurology, the American Congress of Rehabilitation Medicine, and the National Institute on Disability, Independent Living, and Rehabilitation Research. The findings suggest that clinicians should refer patients with PDoC to multidisciplinary rehabilitation teams with specialized training for optimal diagnostic and prognostic evaluations for further management, including effective medical monitoring and rehabilitative care. Prognostic counseling by clinicians should acknowledge that favorable outcomes and prognoses in patients with MCS diagnosed within 5 months of injury and traumatic etiology are variable [14].

Patients with non-traumatic injury exhibit a shorter window of recovery and greater disability than patients with TBI, and a majority of patients with traumatic injury regain consciousness within 12 months, and those with non-traumatic etiology by 3 months [1, 5, 8]. Nevertheless, our results showed that the recovery of patients with non-traumatic etiology may be prolonged. EDoC occurred in 217 (154, 345, range 64–1143) median days after non-traumatic injury and in 158 (124.25, 292.5, range 85–575) median days after TBI. These heterogeneous outcomes may be attributed to the Korean rehabilitation system, which allows intensive neurorehabilitation for both VS/UWS and MCS within 2 years of onset. Similar to previous studies, the prognosis was more favorable and heterogeneous for MCS than for VS/UWS and patients in MCS manifested EDoC in 209 (131.5, 346, range 64–1143) median days compared with 164 (124, 236.25, range 112–345) median days in patients with VS/UWS [11, 12]. Overall, the complexity of recovery outcomes in our study was consistent with recent findings reported in longitudinal studies of PDoC [4, 6–9, 15–19].

From a neurobehavioral perspective, our findings demonstrated that arousal and auditory functions were the most prognostic markers of emergence from PDoC. These findings were supported by higher levels of activation in the auditory association cortex using BOLD functional magnetic resonance imaging (fMRI) in response to a familiar voice speaking the patient’s name, indicating factors associated with better prognosis [14, 20]. Furthermore, this study clinically supports previous reports suggesting that the level of auditory processing revealed by fMRI was strongly correlated with the 6-month outcome in each patient [21]. Di et al. reported earlier that the cerebral response to the patient’s own name uttered by a familiar voice, which was measured with fMRI, might be a useful tool to preclinically distinguish minimally conscious states in a few patients behaviorally classified as vegetative [22].

At the level of functional connectivity, the auditory network is considered the most significant brain parameter distinguishing MCS from VS/UWS [23]. The regions of the auditory network comprising bilateral auditory and visual cortices are functionally connected in MCS more than in VS/UWS. The auditory-visual functional connectivity, also referred to as cross-modal interaction, is related to multisensory integration [24]. Multisensory integration has been suggested as a facilitator in the top-down effects of higher-order regions, which may be necessary for conscious perception [25, 26]. Meanwhile, the cross-modal auditory-visual functional connectivity pairs are preserved in thalamocortical connectivity [27]. Resumption of the functional relationship between thalami and associative cortices, such as prefrontal and anterior cingulate cortices, may lead to the restoration of consciousness, consistent with the behavioral expression indicated by auditory or communication subscales in the CRS-R [28].

In a recently published cross-sectional multimodal imaging study analyzing the neural correlates in patients who emerged from MCS, the patients who emerged from MCS were characterized by a correlation between the networks and increased brain metabolism [29]. Further, novel behavioral correlates of auditory mismatch negativity event-related potentials (ERP) were detected in the auditory cortices [30].

It is worth mentioning that Giacino et al. tracked the recovery of six behavioral benchmarks derived from the CRS-R over a 6-week period during inpatient rehabilitation in patients with traumatic PDoC that extended four to 16 weeks post-injury [31]. The study revealed that patients in MCS with preserved language function were most likely to recover other high-level behaviors associated with functional recovery, analogous to the results in the present study. Moreover, members of the Traumatic Brain Injury Model Systems reported that a substantial number of patients with PDoC admitted to acute inpatient rehabilitation recovered independent functioning over as long as 5 years, especially if they followed commands before hospital discharge [6].

With regard to the temporal dynamics and cumulative recovery outcomes of the neurobehavioral profiles, our study revealed the highest probability of advanced motor function in the first 6 months, similar to early motor recovery in stroke patients, which primarily occurs within the first few months [32]. However, after 1 year, the auditory and communication functions also improved and showed the greatest cumulative probability of improvement in the 2 years post-injury. In this context, when the patient fails to show cortically driven behaviors, such as communication, during the first year after the brain injury, it is important to adopt further powerful approaches to identify cortical activity or ‘volition without action’ based on fMRI, as well as electroencephalography and ERP [33, 34].

Our results should be interpreted cautiously because of the small sample size and the limited number of patients investigated. Further, similar to all retrospective analyses, we could not control the assessment intervals of CRS-R that may have influenced the results. The CRS-R evaluation period varied from daily to every 6 weeks, with an average of monthly assessments. Even though the CRS-R has served as a useful tool for the differentiation between MCS and VS/UWS with high reliability, validity, and sensitivity, spontaneous variability of the relevant neuronal or non-neuronal parameters over time in patients with severe disorder of consciousness may lead to spontaneous fluctuations [35, 36]. Hence, individual variability on the CRS-R may suggest limited diagnostic accuracy. Previous studies have reported high rates of misdiagnosis of PDoC, reaching up to 40% [37, 38].

Several studies have reported the beneficial effect of neuroimaging technologies, such as arterial spin labeling, magnetic resonance imaging, proton magnetic resonance spectroscopy, diffusion tensor imaging metrics, and voxel-based lesion-symptom mapping in the assessment of patients with severe brain injuries [39–43]. Future studies comprising more homogeneous and larger samples, with prospective and regular assessment of CRS-R, combined with neurotechnology-based assessments may corroborate our study findings.

Notwithstanding these limitations, our study facilitates clinician investigations of individuals with PDoC who can potentially benefit from inpatient rehabilitation and the establishment of optimal rehabilitation programs. Indeed, careful observation and evaluation of auditory perception and the facilitation of auditory responses may be important for successful outcomes.

## Conclusions

Significant recovery of consciousness was observed in patients with PDoC during inpatient neurorehabilitation. The course and prediction of the recovery and the effects of neurobehavioral signs on the emergence from PDoC were elucidated in this study. In particular, careful evaluation of auditory perception and facilitation of the auditory response may be clinically important for the successful outcomes of neurorehabilitation in patients with PDoC.

## Supplementary information

**Additional file 1.** Demographic data. Demographic data including age ranges, sex, level of consciousness at admission, etiology, injury type, craniectomy, cranioplasty, CRS-R score at admission, hydrocephalus, presence of VP shunt, anti-epileptic drug, seizure, education, duration of disorder of consciousness, and emergence from DOC are described.

**Additional file 2.** CRS-R scores data. Total score and six neurobehavior domains based on the JFK Coma Recovery Scale-Revised (CRS-R) at admission and at discharge of enrolled patients are demonstrated.

## Data Availability

The datasets used and/or analyzed during the current study are available and deposited.

## Abbreviations

PDoC: Prolonged disorders of consciousness
EDoC: Emergence from prolonged disorders of consciousness
CRS-R: JFK Coma Recovery Scale-Revised
MCS: Minimally conscious state
DoC: Disorders of consciousness
VS/UWS: Vegetative state/unresponsive wakefulness syndrome
ABI: Acquired brain injury
CDW: Clinical Data Warehouse
TBI: Traumatic brain injury
IQR: Interquartile range
HR: Hazard ratio
NA: Not applicable
fMRI: Functional magnetic resonance imaging
ERP: Event-related potentials
